# Six-Month Lower-Leg Sensory Stimulation Augments Neural Network Connectivity Associated With Improved Gait

**DOI:** 10.1093/geroni/igab046.3439

**Published:** 2021-12-17

**Authors:** Chun Liang Hsu, Ikechukwu Iloputaife, Lars Oddsson, Brad Manor, Lewis Lipsitz

**Affiliations:** 1 Hinda and Arthur Marcus Institute for Aging Research, Harvard Medical School, Burnaby, British Columbia, Canada; 2 Hebrew Senior Life, Boston, Massachusetts, United States; 3 RxFunction, Eden Prairie, Minnesota, United States; 4 Hinda and Arthur Marcus Institute for Aging Research, Harvard Medical School, Boston, Massachusetts, United States; 5 Hebrew SeniorLife, Boston, Massachusetts, United States

## Abstract

Foot sole somatosensory impairment associated with peripheral neuropathy (PN) is prevalent and a strong independent risk factor for gait disturbance and falls in older adults. A lower-limb sensory prosthesis providing afferent input related to foot sole pressure distributions via lower-leg vibrotactile stimulation has been demonstrated to improve gait in people with PN. The effects of this device on brain function related to motor control, however, remains equivocal. This study aimed to explore changes in brain network connectivity after six months of daily use of the prosthesis among individuals with diagnosed PN and balance problems. Functional Gait Assessment (FGA) and resting-state functional magnetic resonance imaging were completed before and after the intervention. Preliminary analysis on participants who have completed the study to date (N=5; mean age 76 years) indicated altered connectivity of the sensorimotor network (SMN), frontoparietal network (FPN), and the default mode network (DMN) post-intervention (Z>3.11, unadjusted p<0.05). Participants displayed an average improvement of 5.5 point in the FGA (Minimal Clinically Important Differences>4 for community-dwelling older adults) that was correlated with connectivity changes (unadjusted p<0.05). Specifically, improved FGA was associated with: 1) increased connectivity between the SMN, cerebellum, and occipital cortex; 2) increased connectivity between the FPN, cerebellum, calcarine and intracalcarine; and 3) decreased connectivity between DMN and intracalcarine. These early findings suggest that long-term use of a lower-limb sensory prosthesis may induce neuroplastic changes in brain network connectivity reflecting enhanced bottom-up sensory-attentional processing and suppression of the DMN that are relevant to gait improvements among older adults with PN.

